# Editorial: Green synthesis of metallic and metal oxide nanoparticles with biological applications

**DOI:** 10.3389/fchem.2024.1546838

**Published:** 2025-01-06

**Authors:** Renata Katsuko Takayama Kobayashi, Raghvendra Ashok Bohara, Mahendra Rai, Gerson Nakazato

**Affiliations:** ^1^ Department of Microbiology, Center of Biological Sciences, State University of Londrina, Londrina, Paraná, Brazil; ^2^ Cúram, SFI Research Centre for Medical Devices, University of Galway, Galway, Ireland; ^3^ Nanobiotechnology Laboratory, Department of Biotechnology, Sant Gadge Baba Amravati University, Amravati, Maharashtra, India

**Keywords:** metallic, metal oxide, synthesis, products, health

Nanotechnology plays an increasingly crucial role in the field of healthcare, offering new opportunities for diagnosing, treating and preventing diseases in ways that were previously unthinkable. An important feature about functionalized nanoparticles involves their syntheses and applications (e.g., antimicrobial, antioxidant, diagnostic). In our Research Topic (RT), biological synthesis or green synthesis was approached due to ecological production of these nanoparticles and sustainable practices on development of new products and processes.

Six studies were conducted about this theme with different approaches on synthesis and their applications. All articles emphasized the characterization of nanoparticles produced with traditional techniques resulting in interesting nanoparticles properties in biological applications.

Four studies described the use of nanoparticles as antimicrobials against infectious caused by significant pathogens mainly multidrug-resistant or “superbugs.” Bacterial resistance to antimicrobials is a serious problem for public health. The American economist, O`Neill, predicted that superbugs will kill more people than cancer by 2050. The characteristics of nanoparticles (type, size, zeta potential, encapsulation) set the level of antimicrobial efficacy. So, the type of synthesis and its standardization are direct to new strategies, targets or materials.

Ullah et al. showed that silver nanoparticles (AgNPs) mediated by olive fruit extract (OFE) under sunlight irradiation of only 20 s, resulted in OFE-capped AgNPs (AgNPs@OFE) with suitable characteristics (70 nm and ZP -40 mV) with antibacterial activity (*Escherichia coli*, *Klebsiella oxytoca, Staphylococcus aureus*), including extensively drug-resistant (*Salmonella enterica* serovar Typhi). Another biological effect of these nanoparticles involved antioxidant scavenging potential against H_2_O_2_, adding benefits in these nanoactives.

Tene et al. synthesized graphene oxide (GO)-based composites functionalized with silver nanoparticles (AgNPs) and copper nanoparticles (CuNPs) as potential alternatives to antimicrobials against a Gram-negative (*E. coli*) and Gram-positive (*S. aureus*) bacteria. AgNP syntheses were mediated by *Calendula officinalis* seed extract and CuNPs by ascorbic acid. The GO/AgNPs and GO/CuNPs showed dose-dependent antibacterial efficacy, in which slight nanoparticle aggregation at higher doses reduced efficacy, highlighting the importance of adjusting the concentration of nanoparticles and refining synthesis techniques to improve their effectiveness.

Kalantari and Turner synthetized and characterized gold nanoparticles mediated by ginger extract and curcumin exhibiting an average hydrodynamic diameter of 15 and 10 nm for curcumin-conjugated AuNPs, respectively. The biosynthesis of these nanoparticles revealed the presence of phenolic and aromatic compounds from ginger extract and curcumin, which play a key role in coating the nanoparticles and enhancing their antibacterial activity against *P. aeruginosa*, *E. coli*, and *S. aureus*. The green-synthesized AuNPs demonstrated significant potential for biomedical applications due to their improved stability, attributed to higher surface charge, and the reproducibility of biological synthesis methods. Additionally, the use of ginger extract and curcumin as reducing agents resulted in nanoparticles with uniform size distribution, superior stability, and remarkable antimicrobial properties compared to chemically synthesized AuNPs. The presence of biomolecules in these biosynthesized nanoparticles further suggest enhanced biocompatibility and functionality, crucial factors for biomedical applications.

Blosi et al. investigated how variations in the synthesis conditions influence the physicochemical properties of AgNPs and the antimicrobial activity of the system. By modifying two key parameters that affect the nucleation and growth of the nanoparticles, the researchers found a configuration that improved the antimicrobial activity, especially when the suspension was applied as a coating on cellulose nonwoven fabrics. This increase in antimicrobial efficacy was attributed to the smaller particle size distribution and the synergistic effect between the silver nanoparticles and the hydroxyethyl cellulose (HEC) matrix.

Verma et al. described the biological methods for the synthesis of ZnO NPs and their role as antimicrobial agents in biotic stress mitigation in different crop plants caused by organisms such as bacteria, fungi, viruses, insects, nematodes and mites, which exploit the machinery of host plants. This study also explores the efficacy of ZnONPs and the toxicological implications of their use in agriculture, suggesting a cautious approach to avoid potential toxic effects. This caution is in line with global goals to eradicate hunger, ensure food security and promote sustainable agriculture.

Shahalaei et al. discussed the several advantages of metal nanoparticles (MNPs) in drug delivery systems and genetic manipulation, such as improved stability and half-life in circulation, passive or active targeting into the desired target selective tissue, and gene manipulation by delivering genetic materials. This study showed that due to easy synthesis and their straightforward structure, MNPs offer a promising platform for the binding of targeting ligands on the surface at low core diameter sizes, making them ideal for use in drug delivery systems.

As the studies in our RT demonstrate, functionalized nanoparticles offer immense potential for addressing some of the most pressing challenges in healthcare, particularly in the fight against antimicrobial resistance and improving therapeutic delivery. From the green synthesis of silver, copper, and gold nanoparticles to the innovative use of nanomaterials in different fields, the emerging applications are diverse and promising.

In conclusion, these advancements signal a new era in medical and environmental care, where innovation meets sustainability, offering hope for tackling some of the most challenging health crises of our time. With continued research and collaboration, the future of nanotechnology in healthcare looks brighter than ever, bringing us closer to a world where diseases that once seemed unbeatable may soon be within our reach to cure or control.

